# The dishomeostasis of metal ions plays an important role for the cognitive impartment

**DOI:** 10.1186/1750-1326-8-S1-P13

**Published:** 2013-09-13

**Authors:** Dehua Chui, Huan Yang, Hecheng Wang, Tuo JI, Jia Yu, Shouzi Zhang, Zheng Chen, WeiZhong Xiao

**Affiliations:** 1 Neuroscience Research Institute, Health Science Center, Peking University, Beijing, China; 2 Beijing Geriatric Hospital, Beijing, China; 3 Department of Neurology, Peking University Third Hospital, Beijing, China

## 

Profound synapse loss is one of the major pathological hallmarks associated with Alzheimer’s disease (AD) and might underlie memory impairment. The homeostasis of metal ions plays an important role in health and neurodegenerative disease by influencing cellular biochemical pathways. The disturbance of some metal ions may have cytotoxic effects, which may cause cell death leading to neurodegenerative disorders such as AD. The aim of the present study was to investigate metal concentrations in whole blood from Chinese AD patients with APOE ε4 allele carrier. Concentrations of metals (magnesium, calcium, manganese, iron, cobalt, copper, zinc, selenium, cadmium, mercury and lead) were determined in whole blood by inductively coupled plasma mass spectrometry (ICP-MS) in 40 Chinese people with different Mini-mental state examination (MMSE) score. Normal APP processing could be restored when magnesium was adjusted back to physiological concentration. Our findings suggest that supplementation of magnesium has a therapeutic potential for preventing AD. We observed that Plasma Mg, Zn and Se levels were found to be significantly lower in patients with AD compared with controls. Furthermore, there is a significant negative correlation between manganese and MMSE score. Whereas other metal ions have no association with MMSE score. These result suggests that dishomeostasis of metal ions may involve in the progress of AD pathology, and elevation of brain magnesium exerts substantial synaptoprotective effects in a mouse model of AD and may have therapeutic potential for treating AD in humans.
